# Pandemic-influenced human mobility on tribal lands in California: Data sparsity and analytical precision

**DOI:** 10.1371/journal.pone.0276644

**Published:** 2022-12-14

**Authors:** Esther Showalter, Morgan Vigil-Hayes, Ellen Zegura, Richard Sutton, Elizabeth Belding

**Affiliations:** 1 Computer Science Dept, University of California, Santa Barbara, California, United States of America; 2 School of Informatics, Computing, and Cybersystems, Northern Arizona University, Flagstaff, Arizona, United States of America; 3 College of Computing, Georgia Institute of Technology, Atlanta, Georgia, United States of America; 4 Skyhook, Boston, Massachusetts, United States of America; Eötvös Loránd University, HUNGARY

## Abstract

Human mobility datasets collected from personal mobile device locations are integral to understanding how states, counties, and cities have collectively adapted to pervasive social disruption stemming from the COVID-19 pandemic. However, while indigenous tribal communities in the United States have been disproportionately devastated by the pandemic, the relatively sparse populations and data available in these hard-hit tribal areas often exclude them from mobility studies. We explore the effects of sparse mobility data in untangling the often inter-correlated relationship between human mobility, distancing orders, and case growth throughout 2020 in tribal and rural areas of California. Our findings account for data sparsity imprecision to show: 1) Mobility through legal tribal boundaries was unusually low but still correlated highly with case growth; 2) Case growth correlated less strongly with mobility later in the the year in all areas; and 3) State-mandated distancing orders later in the year did not necessarily precede lower mobility medians, especially in tribal areas. It is our hope that with more timely feedback offered by mobile device datasets even in sparse areas, health policy makers can better plan health emergency responses that still keep the economy vibrant across all sectors.

## Introduction

During the onset of the COVID-19 pandemic in the United States (US), unprecedented preventative measures such as stay-at-home (SAH) and social distancing orders were enacted by local, state, and federal governments to lower the case rates of virus transmission. Despite the apparent success of these orders and increasing compliance with masking and personal distancing through 2020, the case rate per capita among Native American populations in California (CA) was consistently more than twice as high as that of the lowest-affected population group [1]. As late as August 2021, American Indians and Native Alaskans experienced almost 100 more cases per 100, 000 people than the state average while making up only 0.5% of the population [2].

Since March 2020, California state-wide public health orders that nominally reduced human mobility and controlled face-to-face interactions were rarely motivated by data-driven analysis of mobility behaviors. Instead, mobility-restricting decisions were based on aggregate measurements of COVID-19 case totals and growth rates in the preceding days [3–5]. These metrics are inherently delayed from representing the true situation of human movement, and thus COVID-19 transmission, by depending on case records from voluntary testing of symptomatic or asymptomatic individuals. Test results must also be aggregated over geography (counties) and time (days or weeks) to protect sensitive information. Handling this aggregated data requires the assumption that testing was uniform over a county and therefor adequately captured residence location and individual demographics. These assumptions have proven unreliable especially in low-infrastructure areas of the United States [6–8].

Mobile device location data has provided key insights into human behavior in response to the COVID-19 pandemic [9–11]. Public datasets showing human mobility are a promising alternative to weekly case totals for assessing a population’s immediate response to distancing orders [12]. Mobility can be updated daily and correlates both to likelihood for new case infections (e.g., predicting growth rate) and to economic impact on areas with diverse demographics [10]. However, publicly available mobility datasets are highly limited by population representation and the need to aggregate over time and space to preserve anonymity [13]. Common biases are well-known [14] and are most often avoided by dropping areas of low information density from an analysis. Legal boundaries of Native American tribal reservations are often relegated to remote areas and historically struggle with a lack of communications infrastructure, making these areas highly likely to be dropped from mobility analysis. Tribal data sparsity is further compounded by the fact that residents typically have fewer devices contributing to datasets relative to the number of devices per user in more urban and non-tribal areas [15]. However, the need to characterize the spread of COVID-19 and the efficacy of distancing orders has made it essential to understand the non-standard mobility trends in hard-hit marginalized and rural communities.

We address this challenge by exploring census block group (BG) clusters of varyingly sparse mobility data gathered from personal devices in rural, tribal, and urban areas. We demonstrate how two predominant analysis methods, mobility time series aggregation and linear correlation between mobility and COVID-19 case growth, can produce meaningful results in these areas of sparse data so long as decision trade-offs in the calculation process are well-understood. In this paper we make the following contributions:

- We analyze precision ranges on time series medians and Pearson correlations with case growth to characterize sparse mobility data. We find that, as a rule of thumb, these methods are at best half as precise in rural and tribal areas as they are in rural non-tribal areas.
- We employ this precision bound to interpret mobility results in southern California to understand differing responses across rural, urban, and tribal areas to COVID-19-related federal and state distancing mandates throughout 2020.
- We find that mobility through urban tribal areas remained consistently low relative to baseline throughout 2020, despite a high correlation with cases in the surrounding county.
- We find that mobility in all areas of southern California, whether urban, rural, or tribal, correlated less strongly with case growth past the initial first wave of cases through May 2020.

These findings are consistent with similar recent work that concentrates on city-, county-, and state-level trends, discussed in the next section. The remainder of this paper surveys related research, presents our mobility and case datasets along with significant dates that drive our analysis, demonstrates our precision calibration approach, and studies how well these calibrated analyses allow us to characterize mobility through the year in context of case growth and distancing orders.

## Background and related work

Our work addresses multiple urgent questions for which extensive research has already been published in response to the pandemic. In this section we highlight related work that most closely complements this one, and also indicate the breadth of ongoing alternative research directions.

### Mobility as an indicator of future case growth

No one standard metric currently defines “mobility”, but several different metrics have been shown to correspond strongly with case growth, such as home-dwell time [16] or daily distance traveled [17]. Publicly-available datasets collected from mobile device location data ([16–23]) typically use a visit-based metric (such as Google [18] and SafeGraph [16]), a distance-based metric (such as Descartes Labs [17]), or a combination of both (such as Cuebiq [21] or Apple Mobility [22]). Regardless of the exact metric, these datasets are typically aggregated at the level of counties or states and fail to capture important differences arising from urban, rural, or other socio-demographic distinctions. Google’s Community Mobility reports explicitly state their metric is not intended for characterizing differences across urban and rural lines [24].

Mobility at a variety of geographic resolutions (including country, state, county, and city) has been shown to strongly correlate with county-wide COVID-19 case growth, prompting the need for more granular mobility data to map human movement with pandemic spread [25–27] in the future. Device-based mobility tracking has applications in estimating, predicting, and preventing the propagation of COVID-19 in communities around the world [28–31]. Correlation analyses between mobility and case growth must compensate for auto-correlation and inherent seasonality in both raw datasets [32]. Similar to previous research, we use a 14-day average of both mobility and case growth and correlate over periods greater than 100 days to capture the linear correspondence between the broader changes over this time rather than the inherently noisy day-to-day correspondence [33, 34]. Interpreting pandemic spread requires a number of innovative mobility-based metrics [29, 33]. Since publicly available datasets of COVID-19 case counts are most commonly presented at the county level [35], prior attempts to categorize case data differences between urban and rural areas rely on county-level classifications [27, 36, 37] rather than intra-county differences.

Our work considers census block groups (BGs) to distinguish urban, rural, and tribal categories of area inside a county and uses mobility through these category clusters to identify unique trends.

### Mobility and socio-economic demographic factors

Extensive work since 2020 infers associations between mobility and a population’s social attributes. Common difficulties of interpreting such datasets are well-explored [14], but most problems are avoided by using larger-granularity datasets aggregated by state or county. The effect that limitations such as data sparsity or variation in population density have on a final analysis is difficult to quantify meaningfully. However, the disproportionate impact of the pandemic on certain communities has shown that understanding the spread of COVID-19 in areas where mobility data holds these limitations is critical [8, 38].

Established work often explores analytical methods of comparing census block group (BG)-level home dwell time (the amount of time spent in the BG where the device overnighted for the most recent weeks) with various socio-economic attributes concentrated on cities [10, 11]. Another alternative is to cluster BGs into groupings of at least a thousand [32]. In the latter, the authors indicate that health policies need to account for the different abilities of people in these clusters to successfully stay at home, but do not explore methods of accounting for such limitations. Systematically characterizing such disparities for underrepresented minorities in the US remains a field of ongoing exploration [39]. Our work quantifies the amount of variation introduced into calculations from geographical variation of population and sample biases, and finds consistent trends that emerge through the noise.

### Distancing order outcomes and utility of mobile device data

Early research demonstrated that social distancing orders succeeded in reducing wide-scale mobility ([9, 25, 33]). However, subsequent studies have shown that at a more local level, these orders may have increased essential travel or enabled more case exposure in close-knit communities [32, 34]. To date, widespread attempts to characterize and anticipate case growth with human mobility metrics fail to capture the essential behavior differences inherent between urban, rural, and especially Native American communities [40, 41].

Early in 2021 the CA government introduced the Health Equity Metric, a strategy for ensuring counties account for case growth rates in their most at-risk communities before proceeding with order changes [3]. Although this metric attempts to scrutinize case rates in marginalized areas, this measure still depends on more extensive testing after cases have already been transmitted. In contrast, deeper analysis of human mobility from mobile device datasets may have been able to preempt case transmission opportunities [13, 42]. This work examines mobility and case trends in areas that may not be well-characterized by either testing initiatives or wide-scale mobile device data analysis.

## Materials and methods

### Datasets

We draw from four sources of data for this study: (i) a mobile device trajectory dataset aggregated at the level of census block groups (BG) from a location services company called Skyhook; (ii) daily COVID-19 case totals at the level of counties from state health departments provided by the Centers for Disease Control and Prevention (CDC); (iii) population and geographic boundary information for both tribal lands and BGs from the US Census Bureau; and (iv) selected COVID-19 event information and distancing order progression from county and state publications or various news articles. We describe each of these in turn below.

#### Mobility data from Skyhook

Skyhook offers location services to third-party apps that, with user consent, log anonymized records of user mobile device location [43]. An app sends new location requests prompted by service-specific needs such as advertising, geo-fencing triggers, or navigation updates. Requests can be triggered as frequently as once per second. Once triggered, Skyhook’s integrated location service calculates an updated position for that device using GPS, cellular, Wi-Fi, Bluetooth, and LP-WAN networks, as available, on a variety of personal mobile devices. It is compatible with Android, Windows, and iOS operating systems. Skyhook estimates between 1% and 5% of the US population contributes to their dataset, with up to 20% in some urban centers.

Skyhook aggregates location data over BGs for each day of 2020 and includes a unique metric called *bounding box itinerancy*: the diagonal of the bounding box around the total area traveled by any device that appears within the geographic and time aggregation boundary (i.e., each BG and day). This metric approximates the better-known *radius of gyration*, which requires calculating the center of mass of all locations as well as the root mean square of maximum trajectory points. *Itinerancy* is faster to calculate and captures the maximum distance traveled by a device each day, averaged over all devices recorded in a BG during that day. Personally identifiable information, such as typical routes or starting points, is not disclosed.

**Fig 1:**
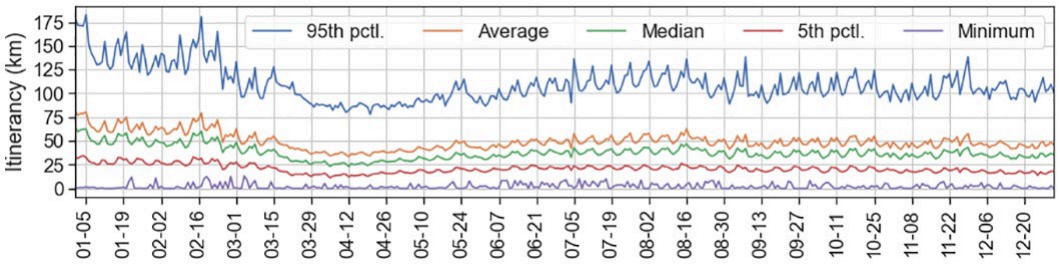
Daily itinerancy aggregation measures in California throughout 2020. Itinerancy averages for each block group are further aggregated over the entire state in five different measures to show the range of variation. Major mobility trends are apparent in all aggregation measures except for the minimum. For the remainder of this analysis, the median is used for mobility except where otherwise noted.

To capture mobility in California (CA), a single aggregate itinerancy entry is added to the dataset for each of the 23, 322 BGs and the 365 days between January 1 and December 31, 2020. These total to more than 8 million entries, each including daily itinerancy average and the number of devices crossing that BG during that day that contributed to the average. Fig 1 shows daily itinerancy over 2020 in CA for five different aggregation measures. Note that major mobility trends are present in both the median and the mean. We use the median itinerancy measure for the remainder of this paper so as not to include skew from uncommon long-distance trips.

We consider *itinerancy* to be a specific metric for measuring *mobility* in general, and in that sense use the terms interchangeably. We later define a *mobility* time series that shows the relative change in *itinerancy* from a baseline set in the first months of 2020; this usage of the term *mobility* is to align our discussion with the widespread convention of denoting general movement changes as a percentage of some baseline. In either case, trends shown through the *mobility* time series are equally evident in the specific *itinerancy* time series. We later use the raw *itinerancy* dataset in some intermediate calculations, but at times continue to refer to it as *mobility* in the general sense.

This paper also deals with specific metrics of dataset *sparsity*. We use this term to refer both to the relatively low (compared to previous work) populations of people in the BGs of interest to this study, and to the varying sizes of device counts that contribute to the Skyhook dataset each day from those same BGs. We also refer to the second factor specifically as *representativity*.

#### Daily COVID-19 case totals

**Fig 2:**
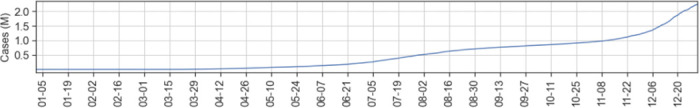
Total daily cases. This total indicates positive test results summed over the entire state from county-level reports each day.

The CDC curates county-level case infection records [44] based on daily updates from state Departments of Health. These numbers represent positive tests reported on that day; unreported cases are not reflected in the data. We examine case infections rather than deaths since we focus this study on understanding the relationship between human mobility and case spread. Daily case totals for CA are shown in Fig 2.

#### Population and geography

Population data for census blocks, census block groups, and counties are provided by the 2018 American Community Survey [45]. Geographic boundaries are sourced from the US Census Bureau [46]. Area calculations use the California Albers projection with EPSG code 3309.

### Key dates

Table 1 shows characteristics of the mobility dataset with breakdowns through regions in CA. We follow region assignments from the state’s COVID-19 health guidance as used for region-wide distancing orders [3]. A set of characteristic time ranges are listed below over which mobility trends in all regions shared relative milestones. Significant event dates are gathered from [47, 48]:

- Post-order (March 19–December 31): The state-wide SAH order on March 19 marks the clearest statewide behavior change, when mobility dropped by about 20% in a few days even after slowly decreasing from baseline during the previous month as individual counties announced local restrictions [47]. The following time ranges are subsets of the Post-order range.
- Dip (April 6–19): these two weeks contain the lowest statewide mobilities. Note that new weekly trends appeared in late March and April where mobility on weekends decreased more than mobility during the week, relative to January–February behavior.
- Rise A (April 20–May 31): Mobility begins to return towards normal, but the strength of the new weekly trends soften around the end of May. Beaches reopened statewide on May 13.
- Rise B (June 1–July 24): Mobility continues to rise cautiously towards normal. New weekly trends return more towards pre-order norms, likely as more businesses started reopening. 51 out of 58 counties reopened most businesses as of June 15, but indoor dining closed again on July 13.
- Summit (July 25–August 20): The highest relative mobility since the SAH occurs in these two weeks in all regions. Schools reopened concurrently or shortly after throughout the second half of August.
- Tiers (August 30–December 31): A new tiered system of tracking, introduced August 28, imposed restrictions on counties depending on positive test rates, case totals from the previous week, and ICU bed capacity [3]. Mobility shows a significant drop and remains about 20% under baseline until the end of the year.

**Table 1:** Characteristics of Skyhook’s BG-level mobility dataset.

Region	Abbreviation	Dates	Days	Block groups (BG)	Samples (M) (% dataset)	Counties (% state total)	Pop. (M) (% state total)
All California	CA	Jan. 1–Dec. 31	365	23,212	8.5	58	39.1
Northern California	NorCal	Jan. 1–Dec. 31	365	539	0.2 (2%)	11 (19%)	0.7 (1.7%)
Greater Sacramento Area	Sacra	Jan. 1–Dec. 31	365	1,830	0.7 (8%)	13 (22%)	2.9 (7.3%)
San Francisco Bay Area	Bay	Jan. 1–Dec. 31	365	5,185	1.9 (22%)	11 (19%)	8.4 (21.4%)
San Joaquin Valley	Valley	Jan. 1–Dec. 31	365	2,458	0.9 (11%)	12 (20%)	4.3 (11.1%)
Southern California	SoCal	Jan. 1–Dec. 31	365	13,200	4.8 (57%)	11 (19%)	22.9 (58.4)

Census block groups (BGs), the basic geographical unit of our dataset, are distributed unequally throughout regions of CA. The Samples column shows the number of records (in millions) for each region. One sample is present for each BG each day of 2020. The bulk of our analysis focuses on Southern California (SoCal), the region with a majority of BGs along with corresponding mobility samples and population.

A final key time period encapsulates the “first wave” of cases, when case growth was rampant before widespread preventative measures were in place. We use the date range February 19–May 31 to define this range.

### Methodology development: Sources of imprecision

**Fig 3:**
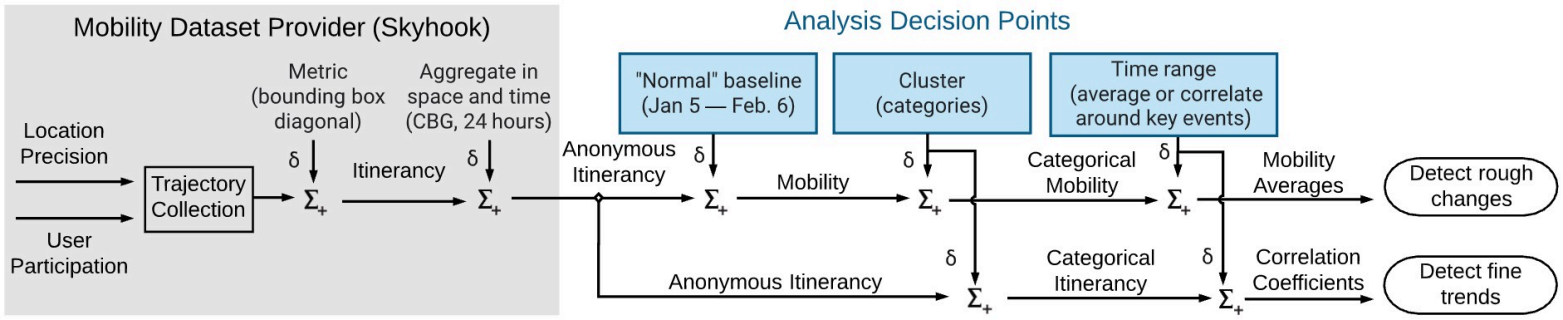
Conceptual diagram of analysis decision points. This paper quantifies how much imprecision can be introduced into final analysis depending on trade-offs at each decision point.

Two analysis methods are immediately useful for understanding mobility in the last year, but are also highly susceptible to the effects of imprecision: grouping mobility time series with medians, and correlating mobility time series with case growth. The data preparation steps for each of these methods are diagrammed in Fig 3.

Many steps in handling mobility data are currently not within researcher control. The accuracy of the location services provider and the number of users contributing to the data are important factors ([14]) that must be taken as-is from the dataset provider. These factors, diagrammed in the gray “Mobility Dataset Provider” box in Fig 3, are outside of the scope of this analysis. Similarly, the choice of specific metric, as well as geographic and chronological aggregation boundaries, are predetermined by the dataset provider. In this analysis we use the dataset from Skyhook described in the Materials section: the metric is itinerancy, the geographic boundary is census block groups, and the chronological boundary is 24-hour periods from midnight to midnight.

Three analysis steps *are* under researcher control and introduce over- or underestimation that can lead to imprecision in final results. These sources are indicated symbolically in Fig 3 as “Analysis Decision Points” by adding some difference *δ* into the calculation flow. While ultimately the accuracy of any analysis is impossible to calibrate without time-consuming ground-truth verification, we can examine how much imprecision is introduced into final results from variation in these decision points.

### Precision trade-off 1: Selecting a baseline to define mobility

**Fig 4:**
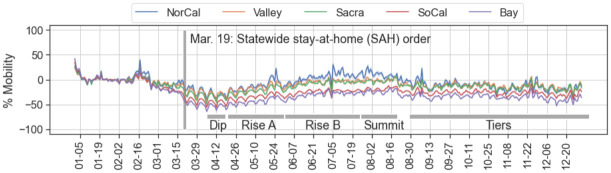
Regional median mobility. Mobility is the percentage of daily itinerancy change from a baseline established in January and February, and is calculated independently for each BG. The median mobility over all BGs in each region is shown here. Higher-population regions exhibit a more radical decrease in mobility than lower-population regions, a pattern that we later show repeats even in more granular clusters of BGs.

Like most publicly available mobility datasets, we use a behavior baseline established in January and February of 2020 and publish daily mobility as the percentage difference in that day’s measurement away from baseline [16, 18, 22]. In Fig 1, a starting date of January 5 is necessary to avoid skewing calculations toward holiday travel around the New Year. General awareness of COVID-19 increased between January 20 and March 20 as case counts grew in CA and counties began enacting emergency measures. Although different baseline end-dates may distort the mobility calculation for any one BG by up to 20%, their effect on aggregate calculations is negligible. We use baseline dates of January 5 through February 6, a range ending about two weeks before COVID-19 warnings became wide-spread. The first local and health emergencies began to be issued in CA counties near the end of this baseline range [47]. Mobility median calculations for the different regions of CA are shown in Fig 4.

### Precision trade-off 2: Selecting cluster thresholds to label BGs on tribal lands

The clustering decision point in Fig 3 defines which BGs will be labeled as tribal or non-tribal and rural or urban. Our method introduces a sliding scale of labels that depend on the percentage that a BG boundary overlaps with a known legal boundary that is already labeled, drawn from population and tribal land boundaries discussed in the Dataset section. By choosing different percentage values to serve as the threshold that defines which label a BG receives, we can experiment with different sizes of clusters of BGs with the resulting label. This serves as a proxy for varying the population and device totals contributing to the dataset. The next decision point compares the sensitivity of mobility analysis while varying clusters by percentage thresholds and points out consistent trends that appear between different cluster types.

#### Labeling threshold variation and data sparsity factors

We assign all BGs two independent labels: either urban or rural, and either tribal or non-tribal. We define a minimum threshold for which a BG geographic boundary needs to overlap a pre-defined rural or tribal boundary in order to be classified as rural or tribal, respectively. By varying this threshold from 1% (least restrictive definition, includes the greatest number of BGs) to 100% (most restrictive, fewest BGs), we can quantify how different geographic clusterings result in varying data density. We then examine data sparsity factors like population make-up and device representativity that appear in the dataset for each cluster. Many other factors impact data sparsity, but these two are important bottlenecks that are quantifiable with the available data. Note that a device that passes through a BG in a day and contributes to the dataset may not belong to a resident of that BG. By varying the overlap threshold by which clusters are defined, this labeling scheme approximates the simultaneous variations of population and device representativity within our dataset.

We select four labeling thresholds to inspect for the remainder of this analysis: 1%, 25%, 50%, and 75%. Using thresholds above 75% reduces the number of tribal BGs to under 20 in all of CA, or less than.01% of the overall dataset, and so the 75% threshold is our maximum.

#### Urban/rural and tribal/non-tribal labeling by threshold

We assign two regional category labels to each BG according to the same threshold cutoff: either U (urban) or R (rural), and either T (tribal) or N (non-tribal). These categories are then combined to show: tribal urban (TU), non-tribal urban (NU), tribal rural (TR), and non-tribal rural (NR) categories.

The US Census Bureau publishes urban/rural assignments to individual census blocks using Tiger/Line geographical files [49]. To label an entire BG, we sum the number of individual blocks within the BG that are assigned the respective label. For a given threshold *t* ∈{1, 25, 50, 75}, if *t*% or more blocks within a BG are labeled rural, then the entire BG in our dataset is labeled rural; otherwise, the BG is labeled urban. For example, in CA when *t* = 50, rural BGs have a median population of ∼1,100 with a max of ∼15,000. Urban BGs have a median at ∼1,500 and a max at ∼39,000.

Tribal boundaries are released every year by the US government and rarely overlap neatly with census boundaries [46]. To approximate clusters of mobility that mostly likely affect residents on tribal lands, we label all data points within a BG as tribal if the BG overlaps tribal lands by *t*% or more. However, some BGs overlap less than *t*% with tribal lands but have a majority of device activity falling within the tribal boundary. The Skyhook dataset includes the daily average device location by latitude and longitude of all devices recorded within each BG each day. If *t*% or more of a BG’s average device locations fall within tribal lands, we label that BG tribal as well.

We then combine the U/R and T/N labels for each BG and consider mobility analyses in each of the combined categories. Of all possible tribal BGs (both TU and TR), 89 of the highest-populated are in SoCal. Only 33 are in NorCal, 6 in the Valley, 4 in Sacramento, and 1 in the Bay area. The remainder of this study focuses on SoCal to take advantage of this majority subset of tribal BGs that all operate under similar regional COVID-19 restrictions throughout the year.

**Fig 5:**
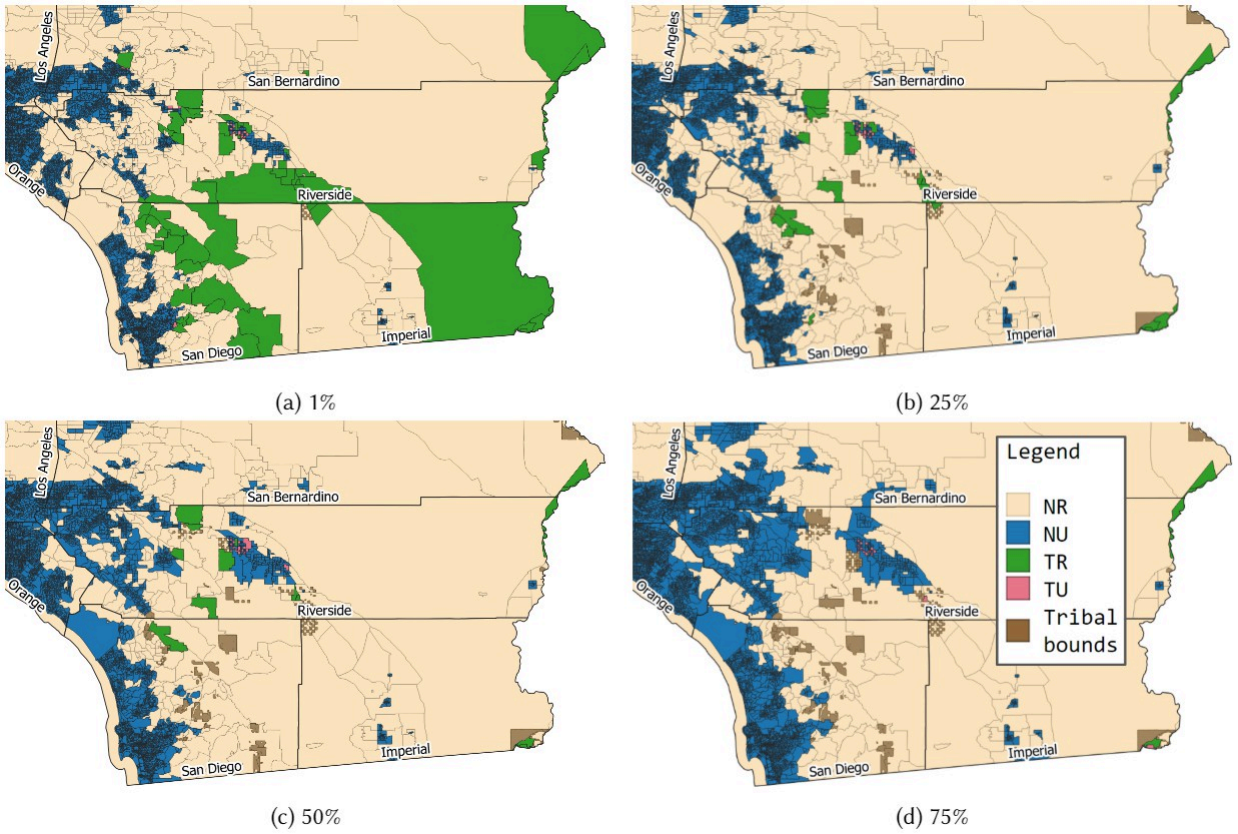
Urban/rural and tribal/non-tribal clustering variations for SoCal counties with tribal presence. BGs in SoCal are labeled as urban (U) or rural (R) and tribal (T) or non-tribal (N) according to the percentage of constituent blocks (for U/R) or area (for T/N) with that label. Shown here are block group labels for four percentage thresholds of constituency, referred to as *labeling thresholds*: 1% (any rural or tribal makeup), 25%, 50%, and 75% (majority rural or tribal makeup). The labels are cross-combined to create the following clusters: Blue areas are non-tribal urban (NU), orange are non-tribal rural (NR), green are tribal urban (TU), and red are tribal rural (TR). This variation in cluster labeling serves as a proxy for varying the underlying BGs that could be assigned each label, subject to different interpretations of the definition of *rural* and *tribal*. Our precision analysis examines how much variation may be introduced into final calculations of rural or tribal mobility depending on the clustering threshold, and considers the corresponding variation in sample size, device density, and population that make up the mobility dataset in each cluster. This figure was generated by the authors from the public census datasets noted in the Materials section [45, 46, 49].

**Fig 6:**
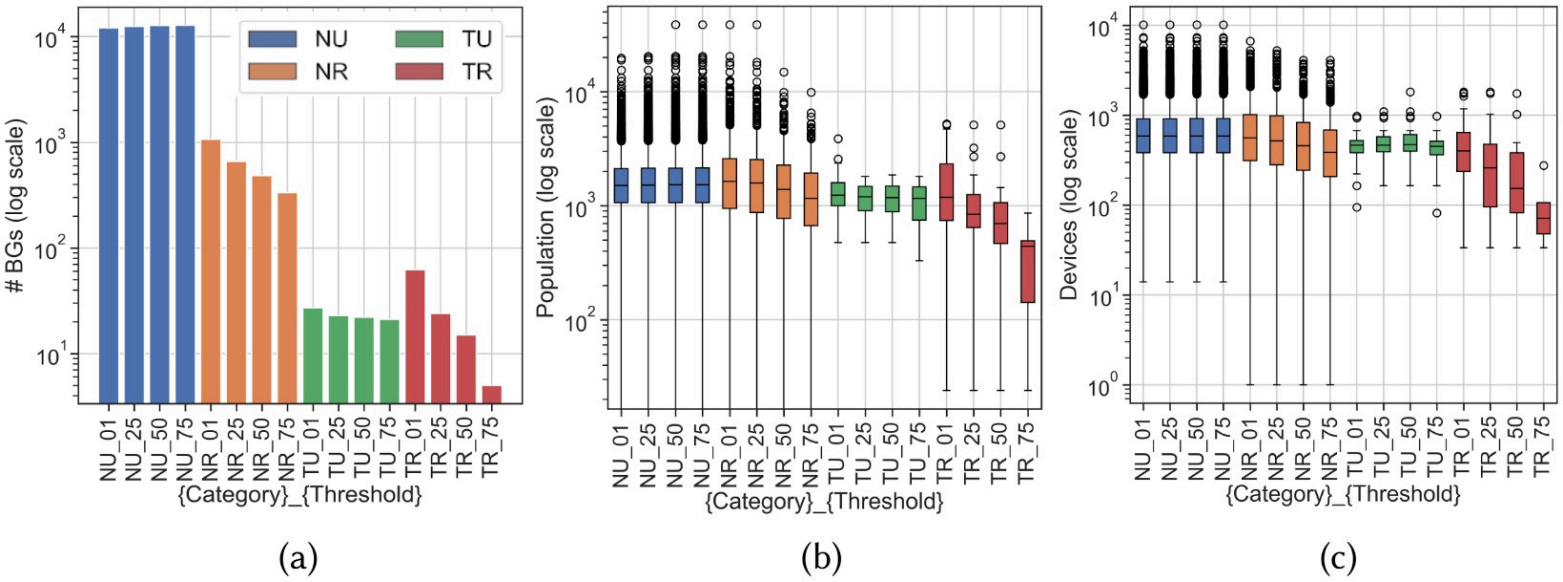
Variation in sparsity factors from labeling scheme thresholds in T/N and U/R category clusters. (a) Block groups appearing in each cluster by different labeling thresholds. The number of BGs labeled rural (R) varies logarithmically depending on threshold cutoffs, while urban (U) BG counts are more linear across thresholds. Tribal BGs show the expected order of magnitude difference from non-tribal, but do not show visible trends at this scale. (b) Populations of clustered BGs vary more widely in rural BGs across clusters than in urban, with non-tribal BGs showing higher outliers. Tribal clusters show greater logarithmic variation than non-tribal. (c) Device counts show similar trends as population, with more linear variation over thresholds for the TU cluster.

Four category clustering schemes for SoCal are shown in Fig 5. BG totals, population ranges, and device count ranges for each category and each threshold cutoff are shown in Fig 6. Unsurprisingly, the non-tribal clusters contain several orders of magnitude more BGs than the tribal clusters as shown on the logarithmic scale of the y-axis in Fig 6a. Similarly, urban clusters contain more BGs than rural clusters except with the least restrictive thresholds on tribal lands (1% and 25%). In Fig 6b, population distributions within each threshold cluster are more consistent across all thresholds in the NU category, whereas the NR category is affected by the restriction on rural BG assignment. Although urban BGs are more numerous, they typically become more geographically concentrated into smaller neighborhoods in more populous areas. Both populations (Fig 6b) and device counts (Fig 6c) are then typically lower for urban areas than for rural areas. These two graphs show that TR clusters experience the most variation across threshold schemes in population and device counts. We examine the degree to which variation in these factors may affect even simple analysis methods to understand mobility in tribal areas during the differing regulation periods throughout 2020.

### Precision trade-off 3: Time ranges for mobility grouping and correlation

#### Analysis method 1: Mobility averages and medians

**Fig 7:**
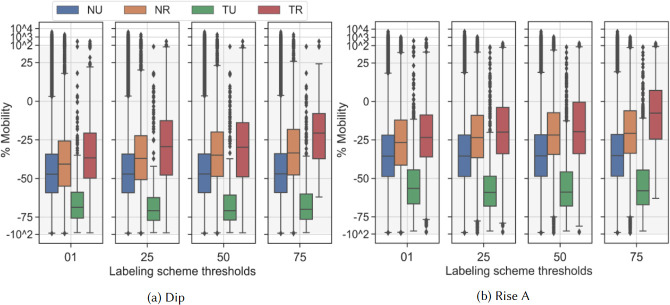
SoCal mobility in categories from different threshold labeling schemes directly following the March 19 SAH. We here use a symmetric logarithm scale to allow positive and negative outliers to be visible while presenting the central values within the shaded linear range (-85, 45). Box extents show the 25^*th*^ to 75^*th*^ percentiles, and whiskers show 5^*th*^ and 95^*th*^. The linear range was chosen to cover the entire whisker extent of all clusters, while allowing outliers to be plotted logarithmically. A comparison of medians across thresholds and categories is shown in Table 2. We explore the relative ranges of mobilities in each cluster across thresholds. The important takeaway is that all labeling thresholds produce similar relative relationships between mobility ranges of clusters, indicating we can expect to see consistent trends between clusters no matter which threshold we choose for analysis.

Our final analysis decision point examines mobility medians in all categories over the labeling schemes during the first wave of cases in SoCal. Fig 7 shows distributions of mobility in SoCal immediately after the March 19 stay-at-home (SAH) order. Fig 7(a) shows the two weeks of lowest mobility (April 5–April 19) and Fig 7(b) shows the subsequent three weeks as restrictions in SoCal slowly began loosening. We refer to these periods as the “Dip” and “Rise A” as defined in the key dates. All labeling schemes confirm that mobility in all categories reached a relative low during the Dip period, then began a return towards baseline during the Rise. Here we see the effects of using different labeling schemes to cluster subsets of BGs that characterize categories. Table 2 presents medians for each mobility category in each labeling scheme over two earliest time periods in the year when new mobility patterns were being established. The tribal rural (TR) category in particular shows a wide range of median results over different labeling thresholds. For the following discussion, we round results in the table to the nearest percentage point. According to the 1% scheme, TR dropped to a median of -34% of its own baseline. According to the 75% scheme, this median drop was only to -17%. Since the 75% scheme is more restrictive than the 1% scheme, it is more likely to include the few BGs that have larger areas with fewer towns. A greater distance between urban centers suggests any travel will be higher within that BG, driving up the average daily itinerancy. Similarly, during the Rise, TR appears to increase up to just under -3% according to the 75% scheme, but only increases to -21% in the 1% scheme. Nevertheless, all labeling schemes show that the tribal-urban (TU) cluster mobility decreased more than any other category, while TR mobility decreased the least in the months following the SAH.

**Table 2:** Median daily mobility across different labeling schemes for cluster categories in SoCal. The “Range” row shows the absolute difference between the lowest percentage point and the highest in each column.

	Dip: April 6–19				Rise A: April 20–May 31			
Labeling Scheme	NU	NR	TU	TR	NU	NR	TU	TR
**1%**	-43.17	-37.67	-63.35	-33.92	-32.92	-25.04	-51.93	-21.52
**25%**	-43.16	-34.01	-63.58	-29.46	-32.84	-21.01	-54.25	-19.16
**50%**	-43.12	-31.68	-62.16	-30.50	-32.75	-19.10	-52.38	-18.26
**75%**	-43.04	-29.54	-62.68	-17.23	-32.62	-17.75	-52.51	-3.25
**Range**	0.13	8.13	0.67	16.69	0.30	7.29	2.32	18.27

This precision analysis confirms our intuition that labeling schemes are a proxy for varying how heterogeneous a dataset might be in sparsity. A mobility average in a category in one county, or region, might involve a different range of populations or representativity. However, no matter the scheme, our results show that relative differences between categories are consistent.

Depending on what classification decision a local health authority uses to define tribal or rural mobility, time-groupings of mobility medians can show differences in conclusive values of up to 18 percentage points: the difference between -34% and -17% in the Dip is 17 points, and the difference between -21% and -3% in the Rise is 18 points. This difference is more than 1/3 of the median mobility drop in all of SoCal during the same time period (∼-47 during the Dip), and so can cause significant interference with interpreting general results in a tribal setting. In contrast, non-tribal rural mobility (NR) only varies by up to 8 percentage points: -37 to -29 in the Dip is 8 points, and -25 to -17 in the Rise is also 8. Non-tribal urban (NU) and TU vary significantly less, but also had almost identical population spreads across threshold schemes. Notably, TU results do not seem dependent on the sample size difference from NU, nor on the variation of device counts between thresholds.

Where previous work has issued general caveats about the effects of population heterogeneity and device sparsity [14], we can now present a quantified observation on precision: *mobility medians in sparse areas near tribal lands may only be half as precise as medians in sparse non-tribal areas*.

#### Analysis method 2: Correlation between itinerancy and case growth

**Fig 8:**
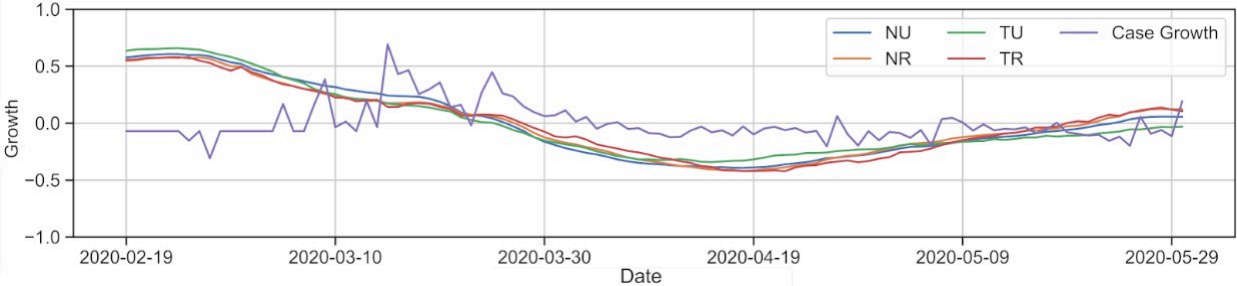
Categorical mobility averages and case growth arrays for all SoCal counties excluding LA and OR. Cluster categories are determined with the 50% scheme. These arrays cover 102 days in the first wave of cases, February 19—May 31. All cluster mobilities share similar seasonality, but our subsequent analysis finds that *tribal* mobility correlation is consistently higher with respect to case growth in the surrounding county.

To explore the relationship between movement changes in an area and subsequent case growth, we perform a Pearson correlation between BG-level itinerancy and county-level case growth similar to the method used in [33]. Case growth is calculated as the natural log of the day-to-day difference of new cases, then averaged over a central rolling window of 14 days. The correlated arrays for itinerancy in cluster categories and case growth are shown normalized in Fig 8. Limitations of this method are discussed in the Background section, but for periods of more than 100 days where we can account for expected seasonality, a 14-day average of both itinerancy and case growth shows the linearity of similar trends. We average itinerancy in cluster categories and compare each category to average county-wide case growth independently.

**Fig 9:**
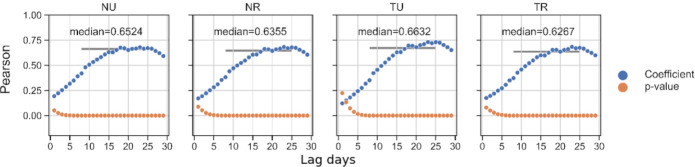
Pearson correlation over lag times. Clusters are shown for all SoCal counties excluding LA and OR, and follow the arrays shown in Fig 8. Lag times capture the possible time between exposure and positive testing. While the maximum correlation coefficient appears for all clusters at 24 days, we include the median of the reasonable lag range (gray bar) suggested by the CDC.

Still following the procedures described in [33, 34], our next step is to inspect the time delay between exposure to the virus and the positive test being entered into the case dataset. This delay time remains uncertain, but guidance from the CDC continues to suggest a waiting period of 14 days is necessary to be certain symptoms will not arise after a possible exposure [50]. We delay itinerancy up to 30 days behind case growth to account for outliers. For each day of lag, the lagged itinerancy and case growth arrays are correlated to produce a Pearson coefficient and *p*-value. We then examine which lag time produces the highest correlation coefficient. Here, our analysis diverges in order to account for the sparsity in our dataset. With sparser mobility data, we find that this peak time is highly susceptible to noise in the correlation vectors. In our experiments, the maximum coefficient uniformly occurs near the latter end of the feasible range of 8–25 days [33, 51]. Fig 9 shows that the highest coefficient for correlations in all cluster types consistently falls at exactly 23 days. This exact maximum coefficient is a product of the similar seasonality of mobility vectors in all clusters. Notably, all coefficients with sufficiently low *p*-values during the entire reasonable lag range tend to fall within 30% of the peak coefficient, and often within 10%. To avoid naïvely assuming the maximum coefficient is as meaningful in sparse datasets as in the more dense datasets that drove this analysis, we instead assume the actual lag days must fall within the reasonable range and simply take the median of all coefficients for lags between 8–25 days whose corresponding *p*-value is ≤0.01. The coefficient median is shown with the gray line spanning the reasonable lag range in all subplots of Fig 9.

We find distinctly different behaviors when considering just Los Angeles and Orange county separately from the rest of SoCal. These two counties contribute a majority of non-tribal urban BGs to the SoCal dataset, but do not include any tribal BGs. We exclude these counties for the moment. Table 3 shows the reasonable median coefficient with two important trends that appear across all labeling schemes: first, mobility through TU clusters consistently correlates more highly than any other category with county-level case growth. Second, rural categories consistently correlate less highly with case growth, for both tribal and non-tribal areas. Interestingly, this result is opposite from a similar study in New Mexico examining case growth and mobility on tribal lands including Navajo Nation [34]. We theorize that the difference is again related to differences in population density, both for Native Americans and in general, between California and New Mexico.

**Table 3:** Median Pearson coefficients across labeling schemes during the first wave.

Scheme	NU	NR	TU	TR
01	0.6512	0.6466	0.6710	0.6483
25	0.6521	0.6430	0.6651	0.6088
50	0.6524	0.6355	0.6632	0.6267
75	0.6526	0.6265	0.6632	0.5720
Range	0.0014	0.0201	0.0078	0.0763

During the first wave, February 19–May 31, the Range column shows the sensitivity of the correlation coefficient to differences in cluster labeling scheme. Tribal areas are more than twice as sensitive as their non-tribal counterparts, while rural areas are orders of magnitude more sensitive than urban.

Increasing the restrictiveness of the labeling scheme reduces the number of BGs in tribal categories and rural categories independently. This sample size reduction produces a stronger effect on the difference between U and R correlation results than on the difference between T and N results. However, Table 3 shows correlation coefficients for TR can vary across labeling schemes twice as much or more as coefficients for NR. NU and TU correlation coefficients are not strongly affected by labeling scheme differences. This consistency is predictable since, as shown in Fig 6, populations and device counts do not vary as strongly across urban schemes as they do in rural. Even though the scale of variation is an order of magnitude smaller in the case of correlation coefficients than in the case of mobility medians, we see a similar pattern: correlation analysis on a sparse mobility dataset in tribal lands in SoCal is at best only half as precise as in the rest of the region.

### Reflections on the utility of precision bounds

The bounds on median and correlation analysis that we have described in the previous section accomplish two purposes. First, they confirm that mobility patterns in the indicated category clusters are distinct and detectable in this dataset despite skew from sparsity. Second, they suggest that mobility calculations in tribal lands are likely to be only half as reliable as calculations in non-tribal lands in SoCal. Future work should calibrate this strategy in other tribal and rural areas across the US to develop quantifiable accuracy bounds on sparse mobility data in these areas.

The first purpose is useful for confirming that the BG-level Skyhook mobility dataset can distinguish unique behavior patterns in tribal, urban, and rural category clusters. Specifically, the sparsity variation plots in Fig 6 show that TR clusters share population and device count attributes with a subset of NR clusters that have lower-than-median values for these factors (see Fig 6a and 6b). Although the median device counts in the TU clusters are quite close to that of NR, median mobility through TU clusters is in the bottom quartile of the NR mobility range. In contrast, TR populations and device counts fall towards the lower half of the corresponding NR ranges, but TR mobility has both a higher mean than NR and a wider range. Although TR and NR mobility characteristics nearly overlap even across labeling schemes, this opposite behavior indicates a significant pattern is present: *mobility in tribal areas during the first wave of COVID-19 cases was distinct from mobility elsewhere in SoCal*. Movement through populated tribal centers was lower than the regional norm, but movement through more remote tribal-adjacent block groups was higher than the respective regional norm. We explore this behavior discrepancy further in the results.

The second purpose of attempting to bound mobility precision through data sparsity is to motivate exploration in future research to better quantify the degree of difference between tribal and non-tribal mobility behaviors. Our results further motivate the need for more accurate pandemic-response datasets in rural and tribal areas. Until further data is available, however, our work prompts continued study of available sparse datasets in other critical areas, such as rural supply lines for food, water, and medicine. Combined with other mobility indicators, such as home dwell time, ability to work or learn from home, and mobility around rural Internet hubs, these factors may provide further insights into how the economic and social disruption through 2020 lead to distinct movement behaviors in marginalized areas.

## Results: Characterizing mobility in California throughout 2020

**Fig 10:**
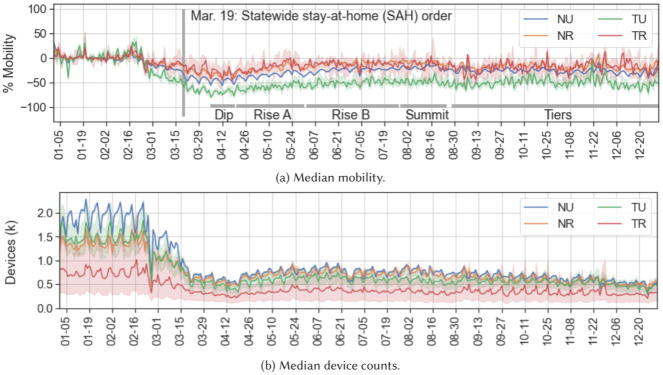
Daily median mobility and device counts seen in each cluster category in SoCal through 2020. Tick marks show every two weeks starting with the first Sunday in January. Ribbons in both (a) and (b) indicate the 5^*th*^–95^*th*^ percentile values of all BGs for each day. Significantly, although device counts in (b) initially diminish in the Dip, they do not track the steady return to baseline seen in mobility in (a) throughout the year.

The precision boundaries explored in the previous section confirm that unique movement trends exist in rural and tribal areas of California, and that our chosen dataset and analysis methods are able to identify them. We can now confidently use these methods to gain insight on the effectiveness of the distancing orders issued throughout 2020 in remote areas of SoCal. Our goal is to discover whether mobility data can show categorical differences in behavior responses to state and local mobility restriction orders. Fig 10 shows median categorical mobility and device counts in SoCal. Since we have established that notable trends are similar in all labeling schemes, for simplicity we use the 50% threshold to define combined categorical clusters in the following results.

The key date ranges described in the Materials section are noted in Fig 10a. These ranges capture major events and visible behavior changes. Fig 10b shows that the median number of devices contributing to the Skyhook mobility dataset also dropped drastically after the SAH and stayed low throughout the year. It is possible that this drop can be partially explained by a reduction in travelers through SoCal, assuming visitors are more likely to travel long distances in a day relative to locals. Note that lower mobility is not necessarily a direct result of lower numbers of devices contributing to the dataset; rather, it is likely that devices that remained in SoCal after the SAH moved around less relative to their own pre-order median. Several other scenarios could also explain this drop, including reduced use of applications that trigger location requests, or devices being turned off entirely. Later in the year the mobility medians for each category eventually returned to within 10% of their pre-order medians, while device counts remained approximately half of their pre-order totals in every category. The exception to this median behavior is in TU clusters, and is discussed further in these results.

TU mobility decreased significantly more, proportional to its own baseline, than mobility through any other area. Rural mobility for both urban and rural BGs dropped the least, with TR medians appearing overall slightly higher than non-tribal rural (NR). We now explore the implications of this trend with respect to county, state, and local COVID-19 regulations throughout 2020.

### Timeline of California restriction and reopening orders

The California Department of Public Health issued a series of distance-related orders that tie closely with our choice of characteristic date ranges for mobility. After the initial SAH order in March, California experienced several additional regulation periods that were intended to limit movement to popular gathering places like restaurants and entertainment facilities. Multiple confounding factors make region-wide mobility trends hard to generalize. First, subsequent waves of cases became less closely tied to mobility as masking and 6-foot distancing became more prevalent [40]. Second, the sequence of restriction and relaxation orders on mobility happened county-by-county, resulting in county-specific movement trends. On August 28, the beginning of the Tier time range, the state government implemented a tiered system of reopening allowances for any county. This system was based on six criteria, including the ability to administer a minimum number of tests and to maintain levels below critical thresholds for both case rates and intensive care unit availability throughout previous weeks [3]. During the Tier time range, SoCal counties were able to independently move through reopening phases coded by a tier color: purple, red, orange, and yellow. The first (and most restrictive) purple tier required a curfew and closures of indoor dining facilities, and it limited travel to essential trips. This caused a general mobility reduction through the remainder of 2020, but allowed different counties to change tiers independently. As cases continued to rise through September and October, additional restrictions were introduced for counties in the purple tier on November 19. These included a limited stay-at-home order that imposed a curfew and limited the sizes of gatherings [4]. On December 3 the residual SAH order was converted to a regional SAH, requiring counties within a region to re-evaluate their tier metrics each week. This order was supplemented on December 6 and December 22 with capacity restrictions on grocery stores and a confirmation that the curfew required in the purple tier was still in place. We note that all of these extensions and adaptations of the original SAH order continued to urge the public to stay home and travel less in order to decrease the likelihood of infectious interactions. However, none of these orders used population movement criteria to determine reopenings or closures; rather, previous weekly averages for tests, cases, and deaths were consistently the main metrics [3].

We inspect median mobility changes in SoCal as a whole until August, and then in individual counties through the end of the year. The continued increase of case rates into 2021 suggests mobility restrictions were only effective when combined with other interventions such as masking and vaccinations. We seek to demonstrate whether these distancing orders were able to accomplish their purpose—to reduce mobility—and if so, whether mobility changed differently in the urban, rural, and tribal BG categories.

### Median mobility changes surrounding key order dates

We select 14 key dates when the following distancing mandates applied to all SoCal counties: issue or extension of a stay-at-home order; closure of private indoor businesses (i.e. bars, dining); and closure of public or private outdoor locations (i.e. public parks, amusement parks, beaches). Mobility medians in different BG categories before and after each order are used to determine the public behavior response to the order. Where dates of significant events were within a week of each other, the event impacting the broader economic sector is used. For example, on June 28, venues that only served alcohol were closed. Just three days later on July 1, all restaurants, wineries, and bars were ordered to discontinue in-door dining. The latter date is selected as the key date. For simplicity, we only consider individual county transitions in Riverside County, which contains the majority of the tribal BGs according to the 50% labeling scheme.

Table 4 shows these key dates, the intent for the order to restrict or relax mobility, the extent of which counties were affected by the order, and the median weekly mobility before and after the order. For these order dates, we find the mobility medians for the two weeks centered around the day of the order. The preceding week includes the day of the order along with the six days immediately before. The following week is the seven days immediately after the order date. Applying this analysis shows that overall, mobility in all categories never dropped as strongly as in the initial March 19 SAH order. As the year progressed, these re-applications of the SAH order appear less and less effective in actually reducing mobility. The effectiveness of these orders on encouraging masking and maintaining a six-foot social distance is outside the scope of this research but is addressed by related research noted in the Background section. Notably, while every relaxation was followed by an increase of mobility, not every restriction was followed by a decrease. The exception to this is Riverside on the purple tier exit on September 29, where the overall and NU median changes are very slightly negative. The low magnitude of these changes suggests that either more local restrictions were in place at the city-level, or that residents in more urban areas had simply not heeded the restriction of the previous week. In rural and tribal areas after this date, mobility did increase more significantly, i.e., by several percentage points rather than by fractions of a percentage. In general, mobility in rural areas decreased less after restriction orders towards the end of the year than earlier in the year. The December 3 Regional SAH was followed by a decrease of less than half the magnitude of the original SAH, except in the TU category. TR clusters also seem to have larger magnitude changes, whether positive or negative, before and after a significant order event, than any other category. Whether this is due to more actual movement through these areas, or just another effect from imprecision, is a question that must be explored with more dense data. A county-specific analysis is necessary to best understand the impact of orders during this time. Similarly, the tier transitions that Riverside County made in October and December accompanied very low magnitudes of decreases and slightly larger increases. The limited and regional SAH orders are further obscured by seasonal tendencies to travel for holidays. While the results in Table 4 cannot tell a complete story, they do suggest how strong or weak an effect distancing orders had on actually controlling mobility. Overall, in the entire SoCal region and in individual counties, our analysis shows that state or county distancing orders did not necessarily succeed in their stated intention to diminish mobility on a week-by-week basis.

**Table 4:** Events expected to influence mobility.

Date	Order type		Extent	% change in mobility				
	Restriction	Relaxation		All	NU	NR	TU	TR
3 / 08	Public Emergency		Riverside	-5.6	-5.6	-3.7	-8.8	-8.5
3 / 12	Parks close		All	-4.9	-5.1	1.2	-9.1	-0.5
3 / 19	SAH		All	-14.8	-14.9	-13.8	-13.4	-4.3
5 / 07		Indoor businesses	All	3.7	3.7	2.9	2.1	6.4
5 / 23		Parks reopen	All	0.5	0.5	0.2	3.7	-8.0
7 / 01	Indoor dining		All	-0.9	-1.0	1.0	-1.2	-2.7
7 / 13	Other indoor activities		All	-0.8	-0.8	-1.2	-1.9	4.4
8 / 28	Tiers enacted		All	-1.9	-1.9	-3.3	-2.1	-0.6
9 / 08	Enter Purple Tier		Riverside	-6.6	-6.6	-5.2	-3.1	-14.9
9 / 29		Exit Purple Tier	Riverside	-0.6	-0.8	0.2	3.5	7.0
10 / 06	Enter Purple Tier		Riverside	-1.4	-1.5	-0.2	1.8	-9.0
11 / 21	Limited SAH		All	4.6	4.5	6.4	-0.3	-5.3
12 / 03	Regional SAH		All	-5.7	-5.7	-7.6	-11.2	-1.6
12 / 21		Exit Purple Tier	Riverside	0.8	0.7	2.2	3.0	-3.2

The “Extent” column defines the counties in SoCal to which the order applied, and the resulting “% change in mobility” applies only to BGs in those counties.

### Mobility correlation with case growth

While distancing restrictions may not clearly create mobility drops, they certainly promote awareness of the importance of masking and keeping personal distance in order to prevent case spread even while gathering. Research emerging from 2020 that studies county, state, and country-level datasets confirms that overall distance traveled correlates less and less strongly with case growth through the year [51]. However, SAH renewals in November and December still relied on distance reduction to ameliorate case spread. Linear correlation over these different time periods can reveal how much the correspondence between mobility and case growth changed throughout the year. We now apply our correlation analysis to identify trends in rural and tribal cluster categories.

**Fig 11:**
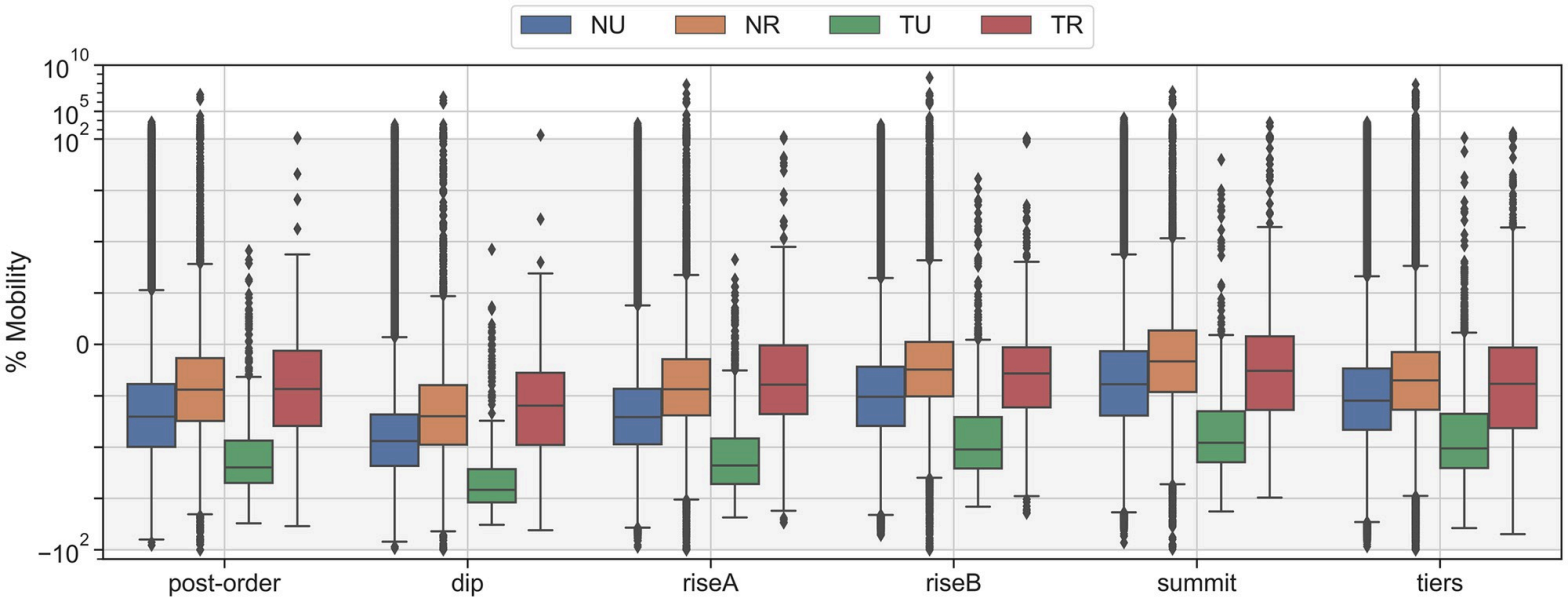
Mobility medians in SoCal during time ranges throughout 2020. All clusters use the 50% labeling scheme to define tribal and rural categories. A symmetric logarithmic scale is used on the y-axis to show a linear scale from -100 to 100, with a logarithmic scale farther out to capture outliers. Relative cluster trends are consistent throughout the year, though NR and TR show nearly indistinguishable distributions in the last months.

Fig 11 shows categorical mobility averages over the characteristic time periods throughout the year. We confirm that all categories remain generally consistent relative to each other, i.e., throughout the year, TU mobility remained the lowest relative to its own January–February baseline, while TR mobility was consistently high and with the largest range of variation. As masking and maintaining personal distance became more prevalent, the correspondence between distance traveled and COVID-19 case growth became less prominent [11].

Table 5 shows Pearson coefficients for the linear correlation between each mobility category and case growth for different periods through the year. The date ranges selected start just before the first wave of cases started to appear, and end after each of the characteristic dates throughout the year. All counties in SoCal are considered together, with categorical mobility and case growth averaged by day across BGs and counties respectively. Los Angeles (LA) and Orange (OR) counties are considered separately against all other counties since these two contribute the largest amount of non-tribal urban BGs (62%) to the overall dataset. The correlations in non-tribal categories of these two counties alone strongly reflect the overall correlation in SoCal, likely due to their majority contribution of NU BGs. The correlation from the remaining NU BGs is presented along with that of tribal categories in the rest of SoCal in the lower sections of Table 5.

**Table 5:** Median correlation coefficients during characteristic time ranges using a 50% labeling scheme for tribal and rural areas.

Category	First Wave (FW)–Rise A Feb. 19–May 31 102 days	FW–Rise B Feb. 19–Jul. 24 157 days	FW–Summit Feb. 19–Aug. 29 184 days	FW–Tiers Feb. 19–December 31 317 days
All SoCal				
NU	0.69	0.60	0.52	0.36
NR	0.67	0.55	0.45	0.32
TU	0.69	0.64	0.59	0.51
TR	0.66	0.57	0.53	0.33
LA & OR only (no tribal presence)				
NU	0.70	0.64	0.54	0.38
NR	0.68	0.63	0.56	0.46
LA & OR excluded				
NU	0.65	0.54	0.45	0.29
NR	0.64	0.51	0.41	0.28
TU	0.66	0.60	0.56	0.48
TR	0.63	0.54	0.50	0.30

**Fig 12:**
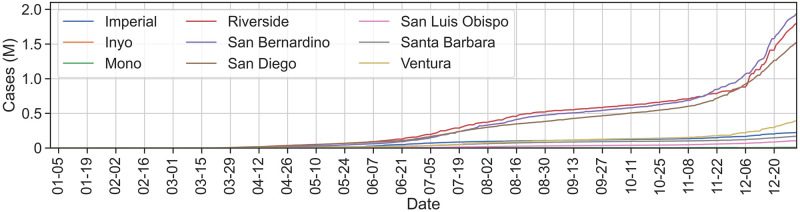
Total cases for all SoCal counties, excluding LA and OR. Case totals are highest for Riverside, San Bernardino, and San Diego counties.

The rows of Table 5 show the differences between categories during the same time range, and columns show differences as the year progressed. Correlation over ranges beginning after the initial mobility drop in March–April produced statistically insignificant *p*-values. To understand how quickly this statistical deterioration happens in each category, we add each characteristic date range progressively to the correlation. While TU coefficients tie with NU in the first wave in all counties, TU maintains the highest coefficient throughout the year in SoCal as well as in just the more rural counties with LA and OR excluded. In Fig 12, we see case totals for these counties continue to rise significantly later in the year.

Interestingly, NR coefficients in all counties and in the rural county grouping remain consistently lowest during all date ranges. In the urban grouping of just Los Angeles and Orange Counties, however, NR shows a stronger correlation once the later date ranges are included. It is possible that in the most urban counties, the BGs that were assigned to the rural category are more similar in populations and device counts to the urban tribal BGs in the more rural counties. While we leave this question to future work, it is likely that a finer granularity of division along urban–rural lines will reveal more consistent correlation distinctions.

We find that TU mobility maintained the lowest median throughout the year but still correlates slightly more highly with region-wide case growth during the first wave than any other category. This finding is consistent with similar analysis in NM around Navajo Nation which found that mobility through high-population tribal areas correlated highly with state-wide case growth [34]. Although the infection rate among tribal and indigenous populations of CA are much lower than the devastating rates in NM, particularly throughout Navajo Nation in 2020 [52], our findings are consistent with the relatively high case rates per capita experienced by CA indigenous peoples relative to most other races [2].

## Conclusion

The use of mobile device location datasets to study a population’s behavior changes in response to distancing orders to combat the spread of COVID-19 is a complex endeavor. Heterogeneity is rampant in datasets capturing mobility, COVID-19 cases, geographic locations of vulnerable populations, and the effectiveness of distancing orders. Mobile device datasets present a unique challenge with irregular population representation and distribution among social strata. However, these datasets are still some of the best sources of information showing real-time movement of people. Understanding population mobility trends is critical for areas of marginalized infrastructure. Our work presents a bounded estimate of mobility in different sparse regions of California. We have demonstrated a controlled variation of sparsity factors such as population density and number of devices contributing to a dataset, and show the resulting range of mobility in coarse- and fine-grained analysis calculations. Overall, our findings show that movement was decreasingly tied to case growth through the year while stay-at-home order renewals were decreasingly effective in controlling movement. We have also shown how useful even fairly sparse data can be in rural areas once precision variation is understood. Our methods reveal distinct behaviors in rural and tribal lands where mobility responses to COVID-19 distancing policies are unique compared to the regional norms. Future work should expand this analysis to other tribal and rural areas of the US. We believe that continued exploration of the accuracy and precision of mobility datasets can help federal, state, and local leaders better react to future public health crises.
